# Environmental Distribution of Pathogenic *Leptospira* spp. in Subtropical Rivers of Japan and Implications for Human Infection

**DOI:** 10.1111/tmi.70157

**Published:** 2026-05-01

**Authors:** Atsuhiro Kanayama, Hiroko Ejiri, Yuki Chiko, Ikumi Namihira, Tomoki Yonaha, Kanetaka Kuba, Miyu Yoshimi, Nobuo Koizumi, Shigeto Takeshima, Koki Kaku

**Affiliations:** ^1^ Division of Infectious Disease Epidemiology and Control National Defense Medical College Research Institute Tokorozawa Saitama Japan; ^2^ Department of General Medicine Okinawa Prefectural Yaeyama Hospital Ishigaki Okinawa Japan; ^3^ Department of Bacteriology I, National Institute of Infectious Diseases Japan Institute for Health Security Shinjuku Tokyo Japan; ^4^ Emergency Department Okinawa Prefectural Yaeyama Hospital Ishigaki Okinawa Japan

**Keywords:** freshwater, heavy rainfall, leptospirosis, river activities, zoonosis

## Abstract

**Objectives:**

Leptospirosis is a zoonotic disease caused by *Leptospira* spp. often linked to freshwater exposure in subtropical and tropical regions. Okinawa Prefecture reports the highest number of leptospirosis cases in Japan based on national surveillance data. Although recreational river activities in Okinawa are a major opportunity for exposure, direct evidence connecting environmental contamination with human infection remains limited. We investigated the spatiotemporal distribution of pathogenic *Leptospira* spp. in rivers on Iriomote Island, Okinawa, a subtropical region with a high leptospirosis incidence.

**Methods:**

Ten laboratory‐confirmed patients treated at the regional hospital and affiliated clinics in 2024 were assessed for demographics, onset dates and exposure histories. Water samples were collected monthly from seven major rivers (June–September and December 2024). Pathogenic *Leptospira* spp. were detected by nested polymerase chain reaction targeting *flaB*, followed by sequencing and phylogenetic analysis.

**Results:**

A cluster of six cases occurred with onset on August 26–27; all reported river activities during incubation periods. In the environmental survey, highly and intermediately pathogenic genotypes were identified in all rivers (*n* = 84 water samples). *Leptospira interrogans* was frequently identified, particularly during summer. Novel sequences within the highly pathogenic group suggest potentially unreported species. Pathogenic *Leptospira* were detected in river water both before and after rainfall. The case cluster occurred following heavy rainfall, suggesting a possible temporal association between rainfall and increased exposure risk.

**Conclusion:**

Pathogenic *Leptospira* spp., including clinically relevant *L. interrogans* , were widely detected in rivers on Iriomote Island. Human case occurrence followed heavy rainfall where pathogenic *Leptospira* were already present in the environment. While this temporal pattern suggests that rainfall may have exacerbated exposure risk, exposures unrelated to rainfall cannot be excluded. This integrated approach may improve case detection and risk assessment in endemic settings and is adaptable to other tropical and subtropical regions with similar exposure risks.

## 1 Introduction

Leptospirosis is a zoonotic disease caused by pathogenic *Leptospira* spp. Humans become infected through contact with freshwater or soil contaminated with the urine of infected animals [1]. Clinical manifestations commonly include fever, conjunctival congestion and myalgia, but may also involve proteinuria, renal failure and jaundice. In severe cases, patients develop respiratory failure, shock, or disseminated intravascular coagulation. An estimated one million cases of leptospirosis occur globally each year, resulting in approximately 60,000 deaths [2]. The risk factors for *Leptospira* infection include contact with animals, exposure to contaminated soil with suitable humidity and pH conditions and farming and other human activities in rivers and wetlands, particularly after floods or typhoons [3, 4, 5]. Recreational and adventure activities in rivers also increase the risk of infection [6, 7]. For prevention, understanding the disease epidemiology in each endemic area is crucial to promote behavioural modifications and personal protective measures.

In Japan, leptospirosis is endemic in Okinawa Prefecture, with approximately 10–40 cases reported annually, which account for approximately half of all cases nationwide. Between 2003 and 2020, 245 cases were reported in Okinawa Prefecture, of which 120 (49%) occurred in the Yaeyama region, including Iriomote Island and 108 (44%) occurred on the main island of Okinawa [8]. Recreational river activities, rather than traditional agricultural work, have recently become the main sources of infection during summer and autumn seasons [9, 10]. However, many aspects of the environmental factors and transmission routes that characterize disease onset in this region remain unclear.

*Leptospira* spp. can be identified and characterized in patients, the environment and animals to help elucidate sources and routes of human infections. The genus comprises 64 species, 17 of which belong to the highly pathogenic P1 clade and others belong to either the intermediate pathogenic P2 or the saprophyte S clades [11]. Among the 43 *Leptospira* isolates from confirmed cases in Okinawa prefecture between 2003 and 2020, the most prevalent was *L. interrogans* serogroup Hebdomadis (46.5%), followed by serogroups Autumnalis (14.0%), Pyrogenes (14.0%), Grippotyphosa (4.7%), Icterohaemorrhagiae (4.7%), Sejroe (4.7%), Australis (2.3%), unidentified serogroup (4.7%) and *L. borgpetersenii* serogroup Javanica (4.7%) [8]. Additionally, an outbreak caused by *L. licerasiae* occurred among U.S. military personnel in 2014 in the northern part of the main island of Okinawa [12].

In the river of Iriomote Island in the Yaeyama region, genotypes belonging to the P1 clade ( *L. kmetyi* , *L. barantonii* and *L. adleri* ) were detected in rivers [13]. However, the genotypes identified in patients in Okinawa Prefecture ( *L. interrogans* and *L. borgpetersenii* ) have not yet been reported in environmental sources, suggesting a lack of evidence directly linking *Leptospira* species in the environment to human infections in this region. From a One‐Health perspective, this study aimed to clarify unknown routes of infection by simultaneously investigating *Leptospira* distribution in rivers and collecting information on potential opportunities for human exposure from confirmed patients. To this end, we investigated the distribution of pathogenic *Leptospira* spp. in rivers on the island and assessed the infection risk by integrating the data with the behavioural history of human cases during the incubation period.

## 2 Materials and Methods

### 2.1 Leptospirosis Case Investigation

Okinawa Prefectural Yaeyama Hospital, located in Ishigaki City, serves as the regional core hospital for the Yaeyama Medical District and provides treatment for patients with suspected leptospirosis, particularly those presenting with moderate or severe symptoms. Two affiliated clinics, Iriomote Seibu Clinic and Ohara Clinic, are located on Iriomote Island and provide primary medical care. Patients were referred to the main hospital as necessary.

Before the environmental investigation, we retrospectively obtained demographic data, onset dates and behavioural histories during the 14‐day incubation period of patients at the hospital and its affiliated clinics who were confirmed to have leptospirosis by laboratory testing at the Okinawa Prefectural Institute of Health and Environment between 2021 and 2023. Concurrent with the environmental investigation, we prospectively collected similar information on confirmed cases at the same institution in 2024.

### 2.2 Ethical Statement

As interviews with patients were required, we consulted the Institutional Review Boards of the National Defense Medical College and Okinawa Prefectural Yaeyama Hospital. We obtained approval (Nos. 4824 and R714, respectively) to ensure that patients would not be placed at any disadvantage. The retrospective study (2021–2023) employed an opt‐out method providing participants with the opportunity to refuse participation. Conversely, the prospective study (2024) obtained written informed consent from all participants prior to their inclusion in the study.

### 2.3 Collection of River Water

**FIGURE 1:**
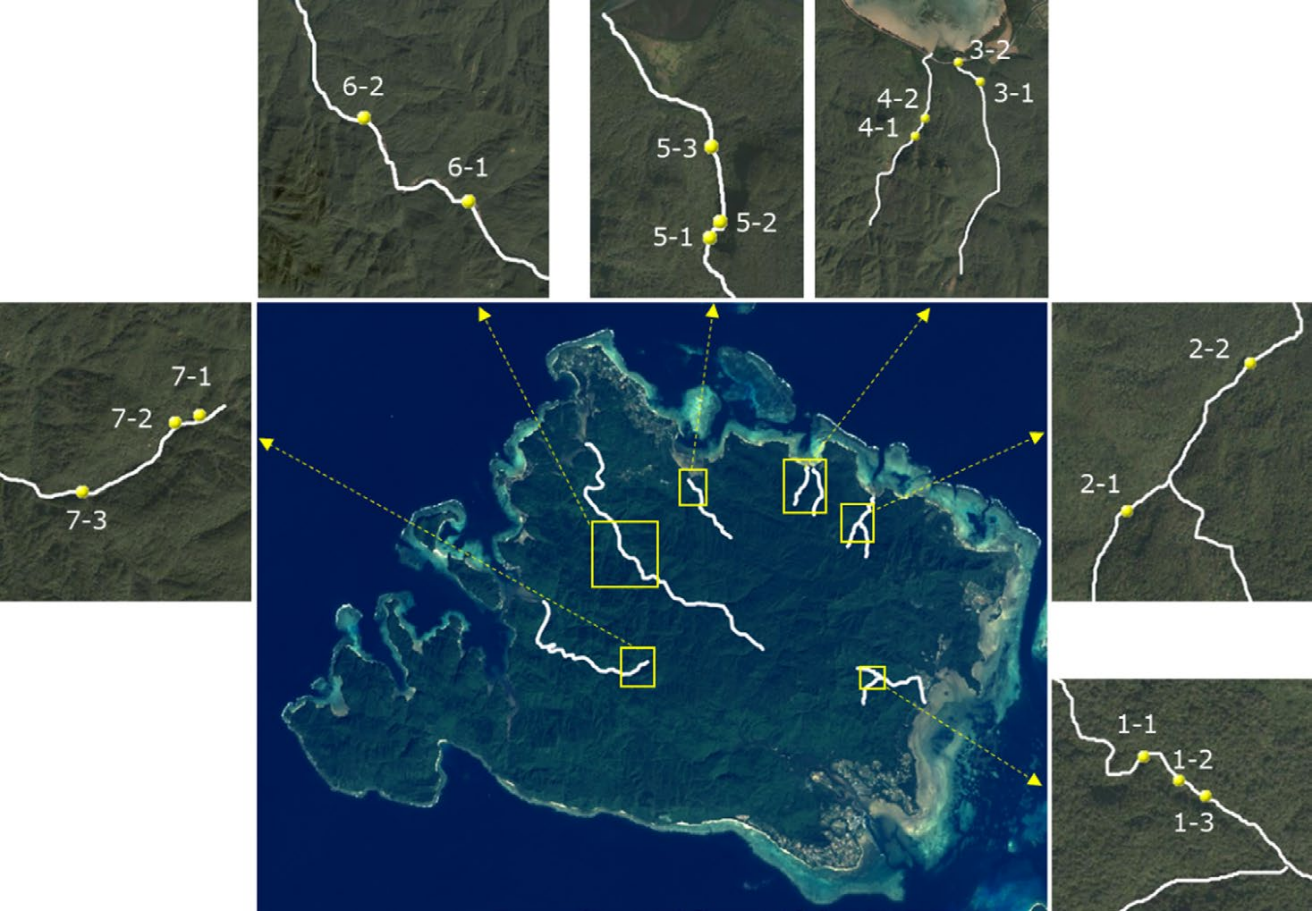
Seven target rivers and water sampling sites on Iriomote Island. Each yellow circle on the enlarged map indicates a water sampling point, numbered by the river followed by the position from upstream.

Two to three sampling sites were established along each of seven major rivers (numbered 1–7) on Iriomote Island, Okinawa Prefecture (Figure 1). Water samples were collected once a month from June to September and again in December 2024. At each site, approximately 1 L of river water was collected in plastic bottles and immediately refrigerated.

### 2.4 Genetic Analyses

Within 3–5 days after collection, 500 mL of each water sample was filtered through a Sterivex filter (pore size 0.45 μm; Merck KGaA, Darmstadt, Germany), and DNA was extracted using the DNeasy PowerWater Kit (Qiagen, Hilden, Germany) [14]. *Leptospira flaB* was amplified by nested polymerase chain reaction (PCR) using specific primers (Table 1). The amplicon size was confirmed by 1% agarose gel electrophoresis. Target bands were excised from the gel and sequenced using the BigDye Terminator v1.1 Cycle Sequencing Kit (Applied Biosystems, Thermo Fisher Scientific, MA, USA) with the second PCR primer sets listed in Table 1.

**TABLE 1:** Primer sequences used in nested PCR.

Name	Sequence	References
*Leptospira* P1_*flaB*_F1	5′‐CTCACCGTTCTCTAAAGTTCAAC‐3′	[14]
*Leptospira* P1_*flaB*_R1	5′‐TGAATTCGGTTTCATATTTGCC‐3′	
*Leptospira* P1_*flaB*_F2	5′‐TGTGGACAAGACGATGAAAGC‐3′	[14]
*Leptospira* P1_*flaB*_R2	5′‐AACATTGCCGTACCACTCTG‐3′	
*Leptospira* P2_*flaB*_F1	5′‐GCAAGCTGTGGATAAAACC‐3′	This report
*Leptospira* P2_*flaB*_R1	5′‐CTGCACGAGTATTTGTTTTGTA‐3′	
*Leptospira* P2_*flaB*_F2	5′‐AGGCAGGCGGAGAGAAATACG‐3′	This report
*Leptospira* P2_*flaB*_R2	5′‐TTTGTAGTTAGCGCAACCAT‐3′	

Abbreviation: PCR, polymerase chain reaction.

The obtained sequences were compared with partial *flaB* sequences of *Leptospira* spp. previously registered in the GenBank database. Nucleotide sequences were aligned using MAFFT in GENETYX software (Nihon Server Corporation, Tokyo, Japan). A phylogenetic tree was constructed using the maximum likelihood (ML) method with 1000 bootstrap replications in MEGA 11 software [15]. Distance matrices of nucleotide sequences were generated using the Kimura 2‐parameter model in GENETYX software. Isolates and NCBI‐registered strains within the same clade (P1 or P2) with a pairwise distance < 0.029 and > 95% sequence identity were defined as having the same *flaB* genotype.

### 2.5 *Leptospira* Culture Isolation

Eight millilitres of river water were inoculated directly into a total of 10 mL Ellinghausen‐McCullough‐Johnson‐Harris (EMJH) broth (Difco, Becton Dickinson, NJ, USA) supplemented with 40 μg/mL sulfamethoxazole, 20 μg/mL trimethoprim, 5 μg/mL amphotericin, 200 μg/mL fosfomycin and 100 μg/mL 5‐fluorouracil [16]. Cultures were incubated at 30°C and examined weekly under a dark‐field microscope. Positive cultures were subjected to single‐colony isolation on EMJH agar plates (1% Noble agar).

### 2.6 Collection of Information on Environmental Factors

Information on the daily rainfall in the study area was obtained from the Japan Meteorological Agency website (https://www.data.jma.go.jp/stats/etrn/index.php).

## 3 Results

### 3.1 Characteristics of Human Leptospirosis Cases

A total of 36 patients with *Leptospira* infection were identified from medical records between 2020 and 2023. Of these, 32 (89%) were male and four (11%) were female, with a median age of 27 years (interquartile range [IQR]: 20–41). The occupations of the affected individuals were as follows: tour guides (*n* = 17, 47%), students (*n* = 8, 22%), farmers (*n* = 1, 3%), others (*n* = 8, 22%) and unknown (*n* = 2, 6%). The months of disease onset were June (*n* = 1; 3%), July (*n* = 5; 14%), August (*n* = 15; 42%), September (*n* = 10; 28%), October (*n* = 4; 11%) and November (*n* = 1; 3%). The outcomes were fatal (*n* = 1; 3%) or recovery (*n* = 35; 97%). The estimated locations of infection were rivers on Iriomote Island (*n* = 23; 64%), Ishigaki Island (*n* = 9; 25%), unspecified locations on Ishigaki Island (*n* = 3; 8.3%) and Yonaguni Island (*n* = 1; 3%). Four of the eight student patients were suspected of representing a cluster with simultaneous exposure at the same river on Iriomote Island.

Among the 10 cases reported in 2024, eight (80%) were male and two (20%) were female, with a median age of 27.5 years (IQR, 21.0–38.5) (Table 2). Nine participants (90%) were tour guides and one (10%) was a student. The months of disease onset were August (*n* = 6; 60%), September (*n* = 3; 30%) and October (*n* = 1; 10%). Common symptoms included fever (*n* = 10; 100%), muscle pain (*n* = 8; 80%) and headache (*n* = 3; 30%). Seven patients (70%) required hospitalization, and all patients recovered. Diagnosis was established by blood PCR in seven patients (70%), urine PCR in six patients (60%), blood culture in five patients (50%) and microscopic agglutination test (MAT) in one patient (10%).

**TABLE 2:** Characteristics of leptospirosis cases identified in the hospital and affiliated clinics in 2024.

		*n*	
Gender	Male	8/10	80%
	Female	2/10	20%
Age	Median (IQR)	27.5 (21–38.5)	
Occupation	Tour guide	9/10	90%
	Student	1/10	10%
Onset	26‐Aug–18‐Oct		
Symptoms	Fever	10/10	100%
	Myalgia	8/10	80%
	Headache	3/10	30%
	Conjunctival congestion	2/10	20%
	Chill	1/10	10%
	Vomiting	1/10	10%
	Renal failure	1/10	10%
	Bleeding	0/10	0%
	Jaundice	0/10	0%
	Proteinuria	0/10	0%
Hospitalization		7/10	70%
Diagnosis	PCR—blood	8/10	80%
	PCR—urine	6/10	60%
	Culture—blood	5/10	50%
	Culture—urine	0/10	0%
	MAT	1/10	10%
Serogroup	Grippotyphosa	4/10	40%
	Hebdomadis	1/10	10%
	Australis	1/10	10%
	Sejroe	1/10	10%
	Unknown	3/10	30%

Abbreviations: IQR, interquartile range; MAT, microscopic agglutination test; PCR, polymerase chain reaction.

### 3.2 Detection of Pathogenic *Leptospira* spp. in River Water

**FIGURE 2:**
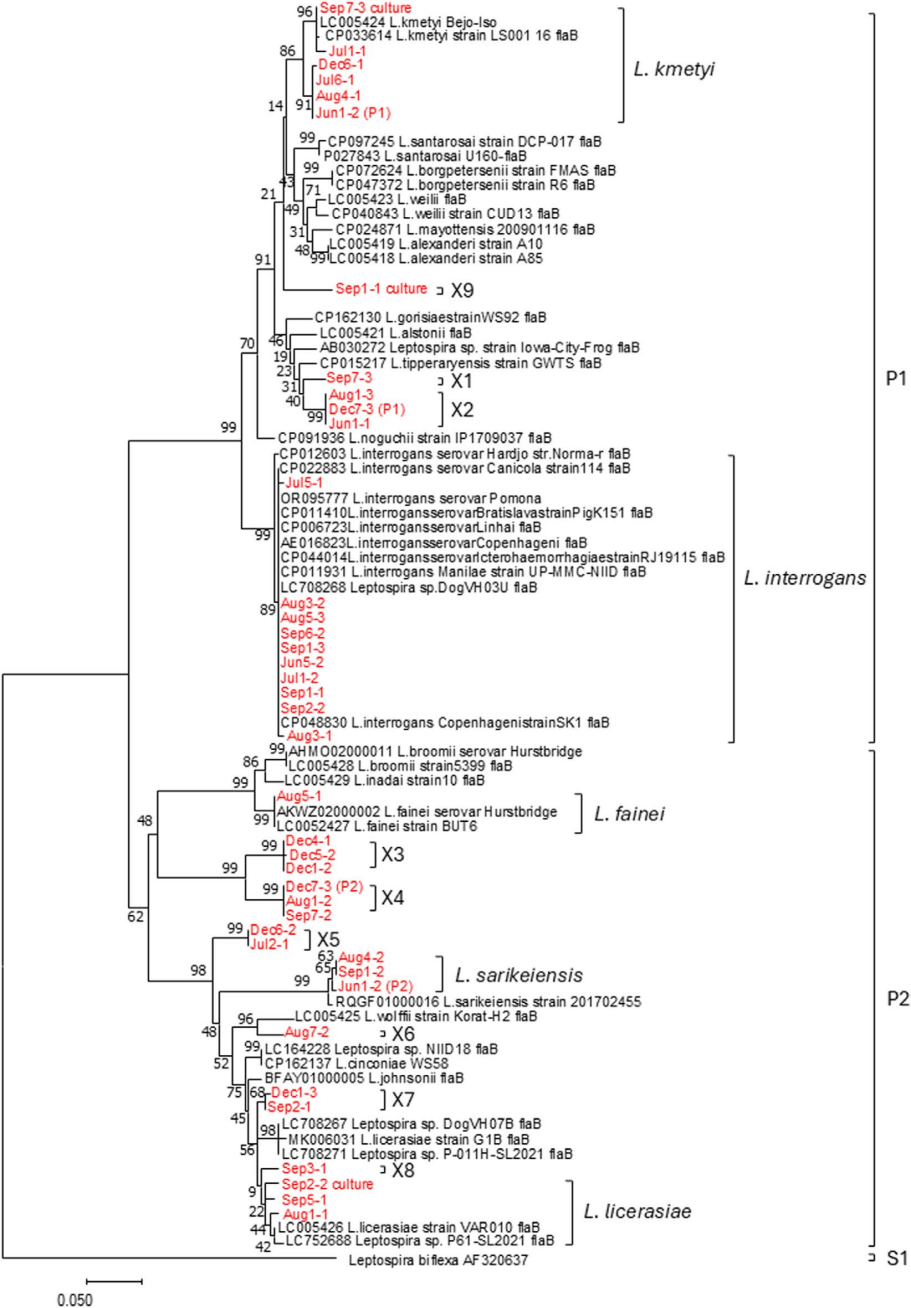
Phylogenetic tree of Leptospira *flaB*. Isolates detected by PCR in this study are shown in red and labelled with the month of collection in 2024 followed by the sampling point (Figure 1). Cultured isolates are labelled as ‘culture’ followed by the corresponding sampling point.

Based on the estimated exposure months and locations from patients up to 2023, water samples were collected from two or three sampling sites along seven major rivers on Iriomote Island in June, July, August, September and December 2024. Among the samples collected from each river, 20%–73% contained *Leptospira flaB* belonging to the P1 clade including *L. interrogans* and *L. kmetyi* while 40%–67% contained *Leptospira flaB* belonging to the P2 clade including *L. sarikeiensis*, *L. licerasiae* and *L. fainei* , as determined by nested PCR followed by sequencing PCR amplicons (Table 3). Furthermore, several gene sequences with < 95% identity to previously reported *flaB* sequences were identified (Table 3). For example, X1 and X2 were genetically distant from all other P1 clade isolates or genotypes, whereas X3–X8 differed from all known P2 clade isolates or genotypes (Figure 2).

**TABLE 3:** Detection of *Leptospira* P1/P2 *flaB* by nested PCR and species identification.

Sampling site^a^	Jun		Jul		Aug		Sep		Dec		Positive (%)	
	P1	P2	P1	P2	P1	P2	P1	P2	P1	P2	P1	P2
1‐1	*X2*	−	*k*	−	+	*l*	*i*	−	−	−	73	67
1‐2	*k*	*s*	*i*	+	+	*X4*	−	*s*	−	*X3*		
1‐3	+	−	+	+	*X2*	+	*i*	+	−	*X7*		
2‐1	−	+	+	*X5*	−	−	−	*X7*	−	−	40	50
2‐2	−	−	+	+	−	−	*i*	+	+	−		
3‐1	−	−	+	+	*i*	−	−	*X8*	−	−	40	50
3‐2	−	+	+	+	*i*	+	−	−	−	−		
4‐1	−	−	+	−	*k*	−	−	−	−	*X3*	20	40
4‐2	−	−	−	+	−	*s*	−	+	−	−		
5‐1	+	+	*i*	+	−	*f*	−	*l*	−	−	47	53
5‐2	*i*	+	−	−	−	+	+	−	−	*X3*		
5‐3	−	−	+	−	*i*	−	+	+	−	−		
6‐1	−	−	*k*	+	−	+	−	−	*k*	−	50	40
6‐2	+	−	+	+	−	−	*i*	−	−	*X5*		
7‐1	+	+	+	+	−	−	+	−	−	−	71	57
7‐2	ND	ND	−	+	+	*X6*	+	*X4*	−	−		
7‐3	+	+	+	+	+	−	*X1*	−	*X2*	*X4*		
Positive (%)	50%	44%	82%	76%	53%	53%	53%	53%	18%	35%	51	52

*Note: X1*–*X8*: *flaB* sequences with < 95% identity to any previously reported *flaB* sequence +: PCR‐positive, species not identified.

Abbreviations: *f*, *L. fainei* ; *i*, *L. interrogans* ; *k*, *L. kmetyi* ; *l*, *L. licerasiae* ; PCR, polymerase chain reaction; *s*, *L. sarikeiensis*.

^a^ Sampling sites on the island are indicated in Figure 1.

The monthly positive rate for both the P1 and P2 clades was highest in July, decreased in the order of August–September and June and was lowest in December (Table 3). *L. interrogans* , the most frequently observed serogroup in clinical settings, was detected in four of the five sampling months (80%) and was prevalent in August and September (Table 4).

**TABLE 4:** Number of *flaB*‐nested PCR‐positive and culture‐positive samples and proportion of positive month by species.

Clade and species	Jun	Jul	Aug	Sep	Dec	Positive month (%)
P1	8	14	9	11	2	100
*L. interrogans*	1	2	3	4	0	80
*L. kmetyi*	1	2	1	1	1	100
*X1*	0	0	0	1	0	20
*X2*	1	0	1	0	1	60
*X9*	0	0	0	1	0	20
Genotype unknown	5	10	4	4	0	80
P2	7	13	9	10	6	100
*L. sarikeiensis*	1	0	1	1	0	60
*L. licerasiae*	0	0	1	2	0	40
*L. fainei*	0	0	1	0	0	20
*X3*	0	0	0	0	3	20
*X4*	0	0	1	1	1	60
*X5*	0	1	0	0	1	40
*X6*	0	0	1	0	0	20
*X7*	0	0	0	1	1	40
*X8*	0	0	0	1	0	20
Genotype unknown	6	12	4	4	0	80

*Note: X1*–*X9*: *flaB* sequences with < 95% identity to any previously reported *flaB* sequence.

Abbreviation: PCR, polymerase chain reaction.

Additionally, water samples collected from each of the seven river sites in September were cultured in parallel with gene identification. After primary culture in the liquid medium, the cultures were spread onto EMJH agar plates, from which three colonies were isolated. The colonies obtained from each plate were confirmed to be identical in their *flaB* sequences, and the resulting strains were designated ‘Sep1‐1 culture’, ‘Sep2‐2 culture’ and ‘Sep7‐3 culture’, corresponding to sampling points 1‐1, 2‐2 and 7‐3, respectively (Figure 2). The first strain had genetically distinct *flaB* sequences from all the other P1 clade isolates and was classified as X9, whereas the second and third strains were identical to *L. licerasiae* and *L. kmetyi* , respectively. Taken together, *L. interrogans* , the most frequently observed species in clinical settings, was predominant in August and September. However, other species were also identified.

### 3.3 Temporal Association Between Human Cases, Environmental *Leptospira* Detection and Rainfall

**FIGURE 3:**
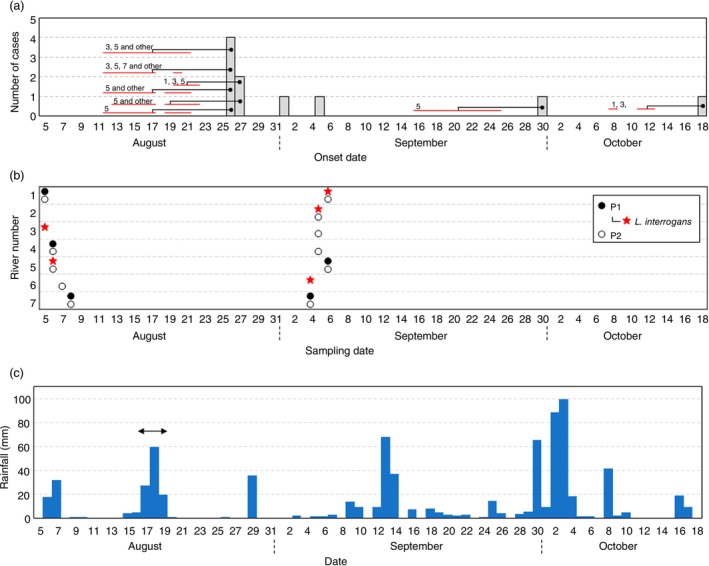
Temporal relationships among onset of human leptospirosis cases, environmental detection of pathogenic *Leptospira* spp., and rainfall on Iriomote Island. (a) Epidemic curve of laboratory‐confirmed leptospirosis cases. Solid red lines indicate dates of river exposure during the incubation period, with river numbers relevant to panel (b) and Figure 1. (b) Detection of *Leptospira* spp. by river and sampling date. The solid black circles represent the P1 clade, the red stars represent *L. interrogans* and the open black circles represent the P2 clade. (c) Daily rainfall. Approaching typhoon No. 9 is indicated by a double‐headed arrow.

Finally, we examined the relationships among the onset of leptospirosis in humans, environmental detection of pathogenic *Leptospira* spp. and rainfall. To identify potential exposures, behavioural history during the estimated 5–14 days incubation period before symptom onset was reviewed (Figure 3a). For the cluster of six patients who developed symptoms on August 26 and 27, the only common behaviour was activity in river No. 5, where *L. interrogans* was detected in water samples on August 6 and the P1 clade on September 6 (Figure 3b). Activities in rivers Nos. 3 and 7 were also confirmed. Other behaviours, such as agricultural work or animal contact, were ruled out. Of the six patients, four were positive for serogroup Grippotyphosa, one was positive for serogroup Australis and one could not be identified.

No information was available regarding which river the patient with onset on September 1 had visited during the incubation period. The patient with onset on September 5 was infected with serogroup Hebdomadis on Ishigaki Island; however, no environmental investigation was conducted. The patient with an onset on September 30 visited only river No. 5, where the P1 clade *Leptospira* (unidentified species) and P2 clade *L. licerasiae* were detected in samples collected on September 6 (Table 3 and Figure 3b). The patient with onset on October 18 was identified as serogroup Sejroe, and the genotypes detected in samples collected in September from rivers No. 1, 3 and 5, where the cases had occurred, were *L. interrogans* (river No. 1), *L. sarikeiensis* (river No. 1) and *L. licerasiae* (river No. 5) (Table 3).

The cluster of six patients who developed symptoms on August 26 and 27 engaged in river activities almost daily before onset (Figure 3a). Looking back at the weather data up to August 12, the earliest day of the average incubation period, heavy rainfall exceeding 100 mm of cumulative precipitation from August 17 to 19 was likely associated with Typhoon No. 9 (Jongdari) (Figure 3c).

## 4 Discussion

This study provides important insights into the public health implications of exposure to pathogenic *Leptospira* from a One‐Health perspective. Clinically significant *L. interrogans* genotypes have been identified in several rivers of Iriomote Island, where the disease is endemic. Our results are consistent with those of previous studies that successfully indicated the environmental sources of *L. interrogans* in the rivers of endemic countries [17, 18, 19]. Furthermore, potentially novel P1/P2 clade genotypes were detected in this study, suggesting the presence of diverse *Leptospira* genotypes in these rivers. Recently, environmental DNA metabarcoding studies have reported *L. interrogans* ‐related genotypes on islands other than Iriomote in Okinawa Prefecture, where fewer human cases than those on Iriomote Island have been recorded [20]. Together, these findings position the rivers of Okinawa as persistent environmental reservoirs of diverse pathogenic Leptospira, providing an essential ecological context for understanding human infection risk in this endemic setting.

In Okinawa Prefecture, serogroups Grippotyphosa and Hebdomadis have been identified in *L. interrogans* from patients [11]. In this study, the serogroup Grippotyphosa was identified in four of the five cases that developed symptoms on August 26 and 27. This contrasts with the reported distribution in the Yaeyama region, where Hebdomadis (53%) typically predominates and Grippotyphosa accounts for only 13.7% [21]. The concentration of Grippotyphosa in this cluster, combined with the fact that River No. 5 was the most commonly visited location, is consistent with a possible shared exposure during this period. However, since other Grippotyphosa patients also visited River No. 3 and River No. 7, where pathogenic strains (P1 clade) were detected, the possibility of infection at these sites cannot be ruled out. The detection of *L. interrogans* at River No. 5 in early August, followed by the detection of the P1 clade at the same river in September (Table 3, Figure 3b), suggests the continued presence of pathogenic *Leptospira* in this environment during the study period. Importantly, pathogenic *Leptospira* were already present prior to the heavy rainfall of August 17–19, indicating that environmental contamination had been established before this event.

While our discrete sampling frequency did not allow us to monitor the immediate pathogen dynamics following the rainfall, the clustered cases occurred after this event. However, our data do not support a direct causal relationship between rainfall and the emergence of pathogenic *Leptospira*. Instead, rainfall may have contributed to increased exposure risk, for example by facilitating the transport of contaminated soil into river water. Exposures unrelated to rainfall events must also be considered.

In this study, multiple leptospiral genotypes across clades P1 and P2 were simultaneously identified by PCR, sequencing and culture isolation from environmental water samples. This suggests that a single animal may shed multiple *Leptospira* genotypes and that multiple animal species may contaminate the same water system. Previous studies have reported that the same host animal can be simultaneously or sequentially infected with multiple *Leptospira* genotypes [22, 23]. In both scenarios, heavy rainfall‐induced water‐level rise could potentially lead to an influx of soil contaminated with pathogenic *Leptospira*, thereby increasing the risk of human outbreak clusters.

Furthermore, human behaviour and exposure frequency significantly influence the risk of human infection. Increased opportunities for water contact during summer leisure activities and tourism can increase the risk of pathogen transmission between humans and animals. Occupations such as tour guides, who are repeatedly exposed to river water for long periods, may be exposed to a series of animal pathogen excretions, thereby increasing the likelihood of outbreaks or clusters.

This study also has some limitations. First, the detection of *Leptospira* spp. was not high‐throughput, and species with low primer affinity for nested PCR targeting the P1 and P2 clades may not have been detected. However, these specific primers likely enabled more frequent detection of the P1/P2 genotypes than did the universal primers used in previous studies. Detection of Leptospira DNA in environmental water samples does not necessarily indicate bacterial viability or infectivity. Nevertheless, the successful culture isolation of pathogenic Leptospira strains from river water in this study provides supportive evidence that viable organisms were present in the environment during the study period. A further limitation of this study is the absence of direct molecular comparisons between environmental isolates and strains obtained from human cases. While species‐level identification from environmental samples and serogroup information from patients suggest epidemiological concordance, these data cannot confirm transmission events at the strain level. To address this gap, future studies should integrate whole‐genome sequencing of paired human, animal and environmental isolates, which would allow definitive tracking of transmission pathways and provide stronger evidence for environmental sources of infection.

In summary, this study demonstrated the widespread presence of pathogenic *Leptospira* spp., including *L. interrogans* , in rivers on Iriomote Island, a region with a high incidence of leptospirosis. By integrating environmental surveillance, molecular detection, clinical epidemiology and meteorological data, we identified a possible temporal association between human infection and heavy rainfall events in a setting where environmental contamination was already present. The observational nature of the study limits our ability to infer causality. Nevertheless, the combined evidence supports a plausible scenario in which heavy rainfall may have increased exposure risk during recreational river activities. From a public health perspective, further enhancement of methodologies for isolating *Leptospira* spp. from environmental sources will strengthen our understanding of transmission between the environment and human/animal hosts [24]. These efforts have significant implications for developing effective countermeasures against occupational exposure. Importantly, this integrated approach may also facilitate improved case detection and risk assessment in endemic areas and could be adaptable to other tropical and subtropical regions with similar environmental and recreational exposure risks.

## Funding

This work was supported by the Japan Society for the Promotion of Science (24K13452).

## Conflicts of Interest

The authors declare no conflicts of interest.

## Data Availability

All sequence data generated in this study have been deposited in the DNA Data Bank of Japan under accession numbers LC906966‐LC907004. The dataset will be publicly accessible at the time of publication.
